# Treatment of hypercholesterolaemia with PCSK9 inhibitors in patients after cardiac transplantation

**DOI:** 10.1371/journal.pone.0210373

**Published:** 2019-01-16

**Authors:** Michael Kühl, Christian Binner, Joanna Jozwiak, Julia Fischer, Jochen Hahn, Alaeldin Addas, Boris Dinov, Jens Garbade, Gerhard Hindricks, Michael Borger

**Affiliations:** 1 Department of Cardiac Surgery, University of Leipzig–Leipzig Heart Center, Leipzig, Germany; 2 Department of Cardiology / Rhythmology, University of Leipzig–Leipzig Heart Center, Leipzig, Germany; Beijing Key Laboratory of Diabetes Prevention and Research, CHINA

## Abstract

**Background:**

Hypercholesterolaemia is common in patients after cardiac transplantation. Monoclonal antibodies that inhibit proprotein convertase subtilisin-kexin type 9 (PCSK9) reduce low-density lipoprotein (LDL) cholesterol levels and subsequently the risk of cardiovascular events in patients with dyslipidaemia. There are no published data on the effect of this medication class on cholesterol levels in patients after cardiac transplantation.

**Methods:**

In this retrospective study we investigated patients who were treated with PCSK9 inhibitors either because of intolerance of statins or residual hypercholesterolaemia with evidence of cardiac allograft vasculopathy. We compared the data of patients prior to the start with these medications with their most recent dataset.

**Results:**

Ten patients (nine men; mean age 58±6 years) underwent cardiac transplantation 8.3±4.5 (range 3–15) years ago. The treatment duration of Evolocumab or Alirocumab was on average 296±125 days and lead to a reduction of total Cholesterol (281±52 mg/dl to 197±36 mg/dl; p = 0.002) and LDL Cholesterol (170±22 mg/dl to 101±39 mg/dl; p = 0.001). No significant effects on HDL Cholesterol, BNP, Creatin Kinase or hepatic enzymes were noticed. There were no unplanned hospitalisations, episodes of rejections, change of ejection fraction or opportunistic infections. Both patients on Alirocumab developed liver pathologies: One patient died of hepatocellular carcinoma and the other developed hepatitis E.

**Conclusions:**

Our study demonstrates that the PCSK9 inhibitors Evolocumab and Alirocumab lead to a significant reduction of LDL Cholesterol in heart transplantation recipients. No effect on cardiac function or episodes of rejections were noticed. Larger and long-term studies are needed to establish safety and efficacy of PCSK9 inhibitors after cardiac transplantation.

## Introduction

Hypercholesterolaemia is common in patients after cardiac transplantation affecting over 90% of patients 5 years post transplantation [1]. The immunosuppressive regime, systemic inflammation and the metabolic syndrome are some factors that are linked to the development of hypercholesterolaemia. Statin therapy has been shown to improve survival of transplanted patients and has been implicated in reducing fatal rejections, decreasing terminal cancer risk and reducing the risk of cardiac allograft vasculopathy (CAV) [2]. It has therefore received a class I treatment recommendation irrespective of cholesterol levels after transplantation [3]. However, some patients cannot tolerate statins or have residual hypercholesterolaemia despite treatment with a statin. Evolocumab and Alirocumab are monoclonal antibodies that inhibit hepatic proprotein convertase subtilisin-kexin type 9 (PCSK 9) and as such are increasing available low-density lipoprotein (LDL) cholesterol receptors on hepatocytes. PCSK9 inhibitors have been shown to not only reduce low-density lipoprotein (LDL) cholesterol levels in patients with hyperlipidaemia [4–6], but also lower the risk of cardiovascular events in patients with established cardiovascular disease [7, 8].

There are no published data on the effect of PCSK9 inhibitors on cholesterol levels in patients after cardiac or other solid organ transplantation. Therefore we sought to determine whether PCSK9 inhibitors are able to reduce cholesterol levels in patients after cardiac transplantation similar to non-transplanted patients. We also planned to assess the frequency of rejection as well as cardiac function during treatment.

## Methods

The Ethic Review Board of the University of Leipzig has approved this study (Local ethical review board number: 399/17-ek). No consent was obtained as the data was analysed anonymously.

All patients who have been treated with Evolocumab or Alirocumab at our institution either because of intolerance of statins or residual hypercholesterolaemia with evidence of cardiac allograft vasculopathy were investigated in a retrospective study. We compared the data of patients prior to the start with Evolocumab or Alirocumab with their most recent dataset on PCSK9 inhibitor treatment.

### Statistical analysis

All continuous values are reported as mean ± standard deviation. The descriptive analysis was performed using the Students’ paired t-test Statistical analyses were performed with SPSS 22.0 software (SPSS Inc, Chicago, IL). A p-value<0.05 was considered statistical significant.

## Results

We identified ten patients with hypercholesterolemia who underwent cardiac transplantation 8.3±4.5 (range 3–15) years ago (S1 Data). The mean age of the patients were 58±6 years and nine patients were men. The most common cause for heart failure was dilated cardiomyopathy in six patients, ischaemic cardiomyopathy in three patients and congenital heart disease in one patient. One of the patients had type 2 diabetes mellitus. No patient had known familial hypercholesterolaemia. Prior to PSCK9 therapy five patients had minor rejections (n = 4 1A, n = 1 1B; ISHLT 1990). Cardiac allograft vasculopathy (CAV) was present in seven patients at baseline. The degree was varying from mild CAV in 2 patients (ISHLT CAV 1) to moderate in 3 patients (ISHLT CAV 2) and severe CAV in 2 patients (ISHLT CAV 3). The immunosuppressive regimen consisted of everolimus in most patients (n = 9, Target level: 4–6 ng/ml), mycophenolate mofetil (n = 6, mean dose: 1000 mg b.i.d.), prednisolone (n = 7; mean dose: 3.75 mg/day), cyclosporine (n = 2; target level: 25–75 ng/ml) and tacrolimus (n = 2, target level 3–5 ng/ml) (Table 1).

**Table 1:** Baseline characteristics.

Baseline characteristics	
**Age—yrs**	58±6
**Male sex—no.**	9
**Time after HTX—mo**	104.3±53.3 (range 42–185)
**Duration of PCSK9 treatment—days**	296±125
**Everolimus—no.**	9
**MMF—no.**	6
**Prednisolon—no.**	8
**Tacrolimus–no.**	2
**Cyclosporin–no.**	2

HTX (heart transplantation), PCSK9 (proprotein convertase subtilisin-kexin type 9), MMF (mycophenolate mophetil)

The reason for PCSK-9 inhibitor therapy (Evolocumab: 140 mg every two weeks n = 8; Alirocumab: 75 mg every two weeks n = 2) was statin intolerance (n = 6) or residual hypercholesterolaemia despite statin therapy (n = 4) (Table 1). We included all patients who were treated with PCSK-9 inhibitors to collect a greater number of patients.

### Therapy effects

**Fig 1:**
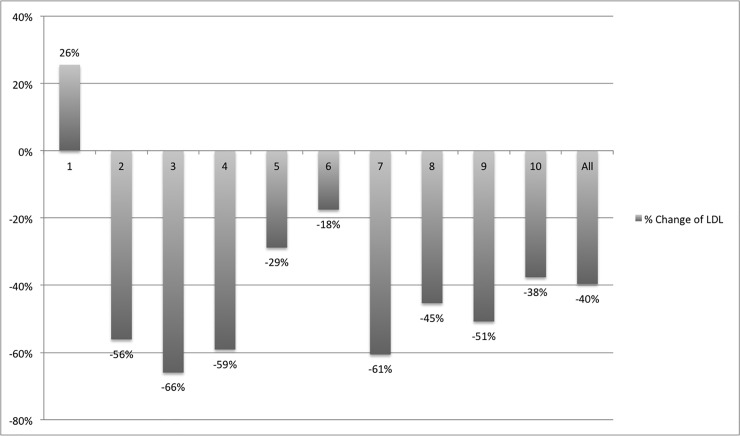
Relative change of LDL cholesterol after PCSK9 inhibitor therapy. Therapy with PCSK9 inhibitors resulted in an overall LDL cholesterol reduction of 40%. As can be seen, the LDL cholesterol levels of individual patients (X-axis) ranged between +26% and -66% when comparing LDL levels after PCSK9 inhibitor therapy to baseline.

The median LDL Cholesterol at baseline was 170±22 mg/dl. The treatment duration was on average 296±125 days and resulted in significant 40% reduction of LDL Cholesterol (101±39 mg/dl; p<0.001). The effect of PCSK9 therapy differed between individual patients and ranged from a 26% increase to a 66% decrease of LDL (Fig 1).

No significant effects on HDL Cholesterol, BNP, fasting glucose, Creatine Kinase or hepatic enzymes were noticed (see Table 2). There were no episodes of rejections, change of ejection fraction, new onset of diabetes mellitus and opportunistic infections (CMV, aspergillosis, candidiasis).

**Table 2:** Treatment effect of PCSK9 inhibitors.

Variable	Baseline	Follow up	P-Value
**Total Cholesterol—mg/dl**	281±52	197±36	**0.002**
**LDL—mg/dl**	170±22	101±39	**0.001**
**HDL—mg/dl**	57±22	60±24	0.451
**Trig- mg/dl**	337±231	255±137	0.301
**Fasting glucose—mg/dl**	118±47	117±48	0.855
**GFR—ml/min/KO**	51±17	52±19	0.827
**ASAT—U/l**	43±38	38±22	0.641
**CK—U/l**	484±1073	451±942	0.495
**CRP–mg/dl**	5.4±5.9	12.87±26.5	0.446
**NT-pro BNP—pg/ml**	828±1540	1326±2142	0.136
**Ejection fraction—%**	59.1±7.2	56±5.5	0.192
**Everolimus level–ng/l**	4.4±1.0	5.4±1.8	0.205
**Tacrolimus level–ng/l**	4.8±0	6.6±1.3	0.875
**Cyclosporin–ng/ml**	72.5±30.4	68.5±10.6	0.822

LDL (low density lipoprotein), HDL (high density lipoprotein), Trig (triglycerides), GFR (glomerular filtration rate), ASAT (aspartate aminotransferase), CK (creatine kinase), CRP (C-reactive protein), NT-pro BNP (N-terminal pro b-type natriuretic peptide)

### Effect on immunosuppression

The levels of the immunosppressants everolimus, tacrolimus and cyclosporine were measured at beginning and at study end. We observed a non significant increase of everolimus and tacrolimus levels as well as a non significant decrease of the cyclosporine levels (see Table 2).

### Adverse events

Both drugs were equally well tolerated. Of the most commonly seen side effects, such as injection site complaints, nasopharyngitis, URTI, allergic reactions, myalgia none were reported by our patient cohort. Two patients however developed liver pathologies: One patient died of hepatocellular carcinoma and the other one developed hepatitis E. Hepatitis E was detected 13 months after initiation of Alirocumb therapy, although the patient had persistently elevated liver enzymes for the last five years prior to starting the PCSK-9 inhibitor.

## Discussion

Our study demonstrates that therapy with the PCSK9 inhibitors evolocumab and alirocumab lead to a significant reduction of LDL Cholesterol in heart transplantation recipients with hypercholesterolemia and therapeutic failure of statin regimens or statin intolerance. No effect on cardiac function or episodes of rejections were noticed in the observed period.

A number of previous studies have demonstrated that Evolocumab is effective in reducing LDL levels by around 60% [4–7, 9, 10] and Alirocumab by around 60% [11, 12]. In our cohort we observed an LDL reduction of 40% (Fig 1). The blunted reduction of LDL levels compare to the reported trials might be explained by the concomitant therapy with steroids and other immunosuppressive medications, which themselves have been shown to lead to dyslipidaemia after transplantation [13] and may counteract the PCSK9 inhibition.

**Fig 2:**
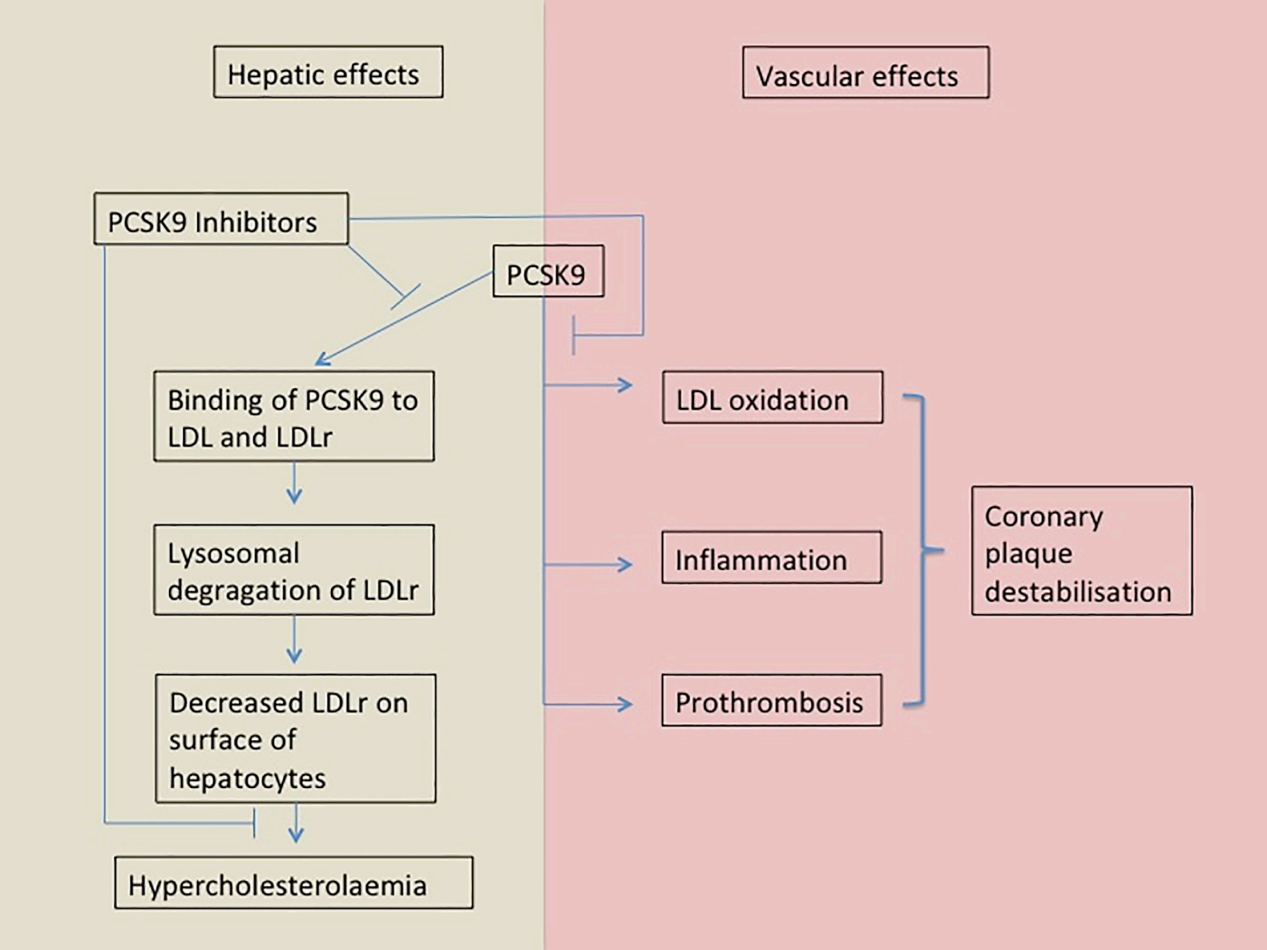
Effects of PCSK9 on dyslipidaemia and the vascular system. By inhibiting the binding of PCSK9 to LDL and LDL receptors (LDLr) more LDLr are expressed on the surface of hepatocytes and thus lead to decreased levels of circulating LDL Cholesterol. Inhibiting the effect of PCSK9 on LDL oxidation, inflammation and prothrombosis treatment could have an effect on coronary plaque stabilisation.

The recent published FOURIER trial compared 13,784 patients receiving Evolocumab with 13,780 patients treated with a placebo drug [7]. The combined primary endpoints cardiovascular death, myocardial infarction, stroke, hospitalization for unstable angina, or coronary revascularization occurred less often in the group treated with Evolocumab (1344 versus 1563, HR 0.85 95% CI 0.79–0.92, p<0.001), but there was no treatment effect on cardiovascular and non cardiovascular mortality as well as hospitalization for worsening heart failure. On the other hand it has been shown that elevated PCSK9 levels and lower LDL receptor (LDLr) levels are associated with adverse outcome in patients with and without heart failure [14, 15]. A possible explanation for this link might be a PCSK9 triggered coronary plaque destabilizing effect through proinflammatory LDL oxidation and direct modification of the plaque composition [16]. PCSK9 is also involved in a direct inflammatory response, largely based on nuclear factor *k*B mediated expression of proinflammatory genes including cytokines, chemokines, and adhesion molecules, but also stimulation of the innate immune system [17, 18]. Monocyte migration capacity as well as inflammatory responsiveness has been shown to be reduced after PCSK9 antibody treatment of patients with familial hypercholesterolaemia [19]. Furthermore, the upregulated nuclear factor *k*B is linked to a prothrombotic state [20] and PCSK9 levels themselves have been shown to correlate with increased platelet aggregation [21] (Fig 2).

There are no reported data on PCSK9 inhibition in patients after cardiac transplantation. However, Shah et al. has published data on the levels of PCSK9 in patients after cardiac transplantation who were switched from a calcineurin based immunosuppressive regime consisting of either tacrolimus or cyclosporine to an mTOR based immunosuppressive regimen with sirolimus at an average of 51 ±30.2 weeks (median, 40 weeks) after transplantation [22]. The switch to sirolimus was associated with an increase of PCSK9 levels (316 ± 105 ng/mL to 343 ± 107 ng/mL; p = 0.041) and a 23% increase of LDL (102.1 ± 37.8 to 125.7 ± 50.6; p <0.002), but there was no evidence that this change of PCSK9 led to a change of lipid levels. Although we did not measure PCSK9 levels in our patients, we could demonstrate that treatment with PCSK9 inhibitors did reduce LDL levels in patients treated with immunosuppression. In our study all but one patient were treated with the mTOR inhibitor everolimus at the time of PCSK9 inhibition. Studies into LDL receptor knockout mice have shown that Cyclosporine therapy leads to a rise in LDL Cholesterol, as well as an elevation of plasma PCSK9 levels suggesting that LDLr mediated lipoprotein clearance plays a protective role against Cyclosporine-mediated hyperlipidaemia and such imply that enhancing LDLr clearance with a PCSK9 inhibitor, might mitigate the effects of Cyclosporin [23]. Whilst, compared to Cyclosporin, therapy with tacrolimus has been shown to result in better lipid profiles in the short term [24–26], after five years however, no significant difference of cholesterol levels between these two immunosuppressive strategies have been reported [27].

Cardiac allograft vasculopathy (CAV) is a common complication after cardiac transplantation with an incidence at the follow-up time points of 1, 5, and 10 years of 8%, 30%, and 50%, respectively [28]. CAV is associated with overall worse outcome after transplantation and risk of sudden cardiac death [29].

The development of CAV is not entirely understood [30]. It is thought that immunological factors, like histocompatibility mismatch, acute rejection episodes and chronic inflammation play an important role in the development of CAV. Nonimmunological factors contributing to CAV include cytomegalovirus infection, age, sex, obesity, diabetes mellitus, hypertension, smoking, ischaemia-reperfusion injury as well as dyslipidaemia [31]. Experiments using a rabbit cardiac transplant model could demonstrate that in cyclosporine treated transplanted mice hyperlipidaemia is linked to the development of fatty intimal proliferation, a precursor of CAV [32]. This observation gives rise to the hypothesis that treating hyperlipidaemia should mitigate the development of CAV. The GLAGOV study was a multicenter, double-blind, placebo-controlled, randomized clinical trial that demonstrated that treatment with Evolocumab led to a 29% reduction of LDL levels as well as a 0.95% (P < 0.001) greater decrease of the coronary plaque volume at 78 weeks compared to placebo treated patients [33]. Three small scale randomized clinical trials assessed the effect of statin therapy on CAV using IVUS [2, 34, 35]. In all trials simvastatin or atorvastation reduced LDL Cholesterol levels and led to a significant decrease of intimal thickness (0.12 +/- 0.07 vs 0.52 +/- 0.17 mm, p = 0.04) [35]. No IVUS was carried out in our patient cohort.

A recent retrospective analysis of 402 patients 8.9 years after heart transplantation demonstrated that patients who were converted from a calcineurin based immunosuppression to the mTOR inhibitor sirolimus experienced a significant attenuation of the progression of plaque volumes (2.8 ± 2.3 mm^3^/mm vs. 0.46 ± 1.8 mm^3^/mm; p < 0.0001) as well as mortality (adjusted hazard ratio: 0.47; 95% confidence interval: 0.31 to 0.70; p = 0.0002) and CAV related events (adjusted hazard ratio: 0.35; 95% confidence interval: 0.21 to 0.59; p < 0.0001).These outcomes were more pronounced in patients that were converted earlier than 2 years after heart transplantation. There was a trend towards higher cholesterol levels in the sirolimus group (192.9 ± 50.6 mg/dl vs. 181.2 ± 45.8 mg/dl; p = 0.06; p = 0.10 after adjustment for baseline measures), but dyslipidaemia was not correlated to adverse clinical outcome [36].

In a pooled analysis of available data from clinical trials the overall rate of adverse events in 12,200 patients with either PCSK9 inhibitors (Evolocumab and Alirocumab) were similar to placebo therapy[37]. The most common side effects were reported to be local injection-site reactions. Abnormal liver enzymes were less common in the PCSK9 groups compared to the placebo group.

One of the patients in our trial developed hepatocellular carcinoma and died subsequently from the disease. He had been receiving PCSK9 treatment for 13 months prior to his death. Elevated PCSK9 levels are linked to the development of hepatic fat accumulation [38] and even to the occurrence of human hepatocellular carcinoma (HCC) [39]. On the other hand, liver tissue of patients with HCC demonstrates decreased expression of PCSK9 levels [40]. Therefore, targeting PCSK9 has been proposed as a new treatment strategy in patients with HCC [41].

The detection of Hepatitis E in one patient treated with Alirocumab was based on persistently elevated liver enzymes and new upper quadrant pain. Other forms of hepatits (A, B or C) were all negative. The dose of mycophenolate mofetil was decreased since the detection of the hepatitis E virus and the patient has been treated conservatively since then. There has been no reports in the literature linking the therapy with Alirocumab with the development of Hepatitis E. However further studies are needed to exclude HCC and Hepatitis E as serious adverse events after the initiation of therapy with Alirocumab.

The results of this study are limited by the nature of the retrospective design, the absence of IVUS data and the small patient number. Although PCSK9 levels were not obtained to demonstrate a treatment effect the significant LDL reduction demonstrates that patients adhered to the therapy regime.

## Conclusions

We demonstrate for the first time that treatment with the PCSK9 inhibitors Evolocumab and Alirocumab achieve a reduction of LDL cholesterol in patients following cardiac transplantation. There were no incidences of acute rejections and the ejection fraction remained unchanged over the observation period. One patient died following treatment with Alirocumab from the consequence of advanced HCC. Larger and long-term studies are needed to establish safety and efficacy of Evolocumab and Alirocumab after cardiac transplantation with a special focus on the development or progression of CAV, as well as to monitor for possible hepatic complications.

## Supporting information

S1 Data The original data is attached as supporting information. (XLS)
